# Melioidosis in Bangladesh: A Clinical and Epidemiological Analysis of Culture-Confirmed Cases

**DOI:** 10.3390/tropicalmed3020040

**Published:** 2018-04-09

**Authors:** Fazle Rabbi Chowdhury, Md. Shariful Alam Jilani, Lovely Barai, Tanjila Rahman, Mili Rani Saha, Md. Robed Amin, Kaniz Fatema, K. M. Shahidul Islam, M. A. Faiz, Susanna J. Dunachie, David A. B. Dance

**Affiliations:** 1 Centre for Tropical Medicine and Global Health, Nuffield Department of Medicine, University of Oxford, Oxford OX3 7BN, UK; susie.dunachie@ndm.ox.ac.uk (S.J.D.); david.d@tropmedres.ac (D.A.B.D.); 2 Mahidol Oxford Tropical Medicine Research Unit, Mahidol University, Bangkok 10400, Thailand; drmafaiz@gmail.com; 3 Peter Medawar Building for Pathogen Research, University of Oxford, Oxford OX1 3SY, UK; 4 Department of Medicine, Bangabandhu Sheikh Mujib Medical University, Dhaka 1000, Bangladesh; 5 Department of Microbiology, Ibrahim Medical College, Dhaka 1000, Bangladesh; jilanimsa@gmail.com; 6 Department of Microbiology, BIRDEM General Hospital, Dhaka 1000, Bangladesh; barai_lovely@yahoo.com (L.B.); tanjila_lo@yahoo.com (T.R.); milisaha77@yahoo.com (M.R.S.); kmshahid2000@yahoo.com (K.M.S.I.); 7 Department of Medicine, Dhaka Medical College, Dhaka 1000, Bangladesh; robedamin@yahoo.com; 8 Department of Critical Care Medicine, BIRDEM General Hospital, Dhaka 1000, Bangladesh; drkanizfatemasb@gmail.com; 9 Dev Care Foundation, Dhaka 1205, Bangladesh; 10 Lao-Oxford-Mahosot Hospital-Wellcome Trust Research Unit (LOMWRU), Vientiane 0100, Laos; 11 Faculty of Tropical Medicine and Infectious Diseases, London School of Hygiene and Tropical Medicine, London WC1E 7HT, UK

**Keywords:** melioidosis, *Burkholderia*, Bangladesh

## Abstract

Melioidosis is known to occur in Bangladesh, but there are few reports about the condition in the published international literature. We set out to review all known cases of melioidosis in the country to date, using both retrospective and prospective data. A web-based literature search was conducted to identify all published case reports, original articles and conference abstracts. Cases were also included from a prospective study conducted in 2017. Fifty-one cases were identified between 1961 and 2017. Cases have been reported from sixteen out of the 64 districts of Bangladesh. The median age of the patients at presentation was 45 years (IQR 37–52), with a significant male (77%) predominance. Many patients (14/39; 36%) were farmers and 83% had diabetes mellitus. A skin/soft tissue abscess was the most common primary clinical presentation (13/49; 27%), followed by septic arthritis (10/49; 20%), pneumonia, and a deep-seated abscess/organ abscess (7/49; 14%). The major challenges to the diagnosis and treatment of melioidosis in Bangladesh are the lack of resources and the lack of awareness of melioidosis. Capacity development programs are urgently required to define the burden of disease and to tackle the mortality rates.

## 1. Introduction and History of Melioidosis in Bangladesh

Melioidosis is an important cause of infectious disease across Southeast Asia, and it is believed to be a problem in Bangladesh, based on anecdotes and a few case reports [1,2,3]. The disease is caused by a highly pathogenic, soil-borne, Gram-negative bacterium, *Burkholderia pseudomallei* [4]. Bangladesh is an example of a highly populous, agricultural country where melioidosis may be a significantly underdiagnosed cause of infection and death. A lack of awareness among microbiologists and clinicians and a lack of diagnostic microbiology infrastructure are factors that are likely to lead to the underreporting of melioidosis. The first reported, confirmed case of melioidosis that was acquired from Bangladesh (at that time known as East Pakistan) was a British sailor, who was travelling east of Suez [5]. In October 1960, his ship was carried half a mile inland near Chittagong by a cyclone, and was deposited in a paddy field [5]. The crew stayed there for three months and were repatriated in January 1961. The patient developed symptoms in May in the UK of that year and eventually received a diagnosis of melioidosis based on pus cultures in Liverpool [5]. Several individual case reports/series of melioidosis have been published from Bangladesh since that time. Cases have also been reported among Bangladeshi immigrants presenting in other countries [5,6,7,8,9,10,11,12,13,14,15,16].

The clinical presentation of melioidosis is widely varied, and a definitive diagnosis requires a skilled microbiology laboratory, making it more difficult to diagnose in low-resource settings such as Bangladesh. Agriculture is the most productive sector of the country’s economy, contributing about 30% of the nation’s GDP and providing over 90% of Bangladesh’s rural employment [17]. A recent modelling study predicted a melioidosis burden of nearly 17,000 cases and 9500 deaths a year in Bangladesh [3,18]. This review identifies 51 cases covering all the published case reports, series, and unpublished cases during the period from 1964 (the case report of the 1961 British sailor) to 2017, with the aim of generating further evidence to increase the awareness of the disease in the national and international healthcare community.

## 2. Review of Melioidosis Cases and Presence of *B. pseudomallei* in Bangladesh

This was a descriptive study involving both retrospective and prospective data analysis. A web-based literature search was conducted using PubMed, Google Scholar, Medline, ResearchGate, and the Bangladesh Journals Online (BanglaJol) database to identify all published, culture-confirmed case reports of melioidosis in Bangladesh. The key search terms were ‘melioidosis’, ‘*Burkholderia*’, and ‘Bangladesh’. Additional published cases were also identified from the personal EndNote database of one of the authors (D.A.B.D.). We also conducted a prospective study during 2017 in Dhaka to identify cases among patients admitted with an acute febrile illness and included patients confirmed as having culture-positive melioidosis, in this review.

### 2.1. Published and Unpublished Cases

Overall, we identified 25 case reports/case series, one original article, and three conference abstracts describing cases of culture-confirmed melioidosis in Bangladesh. Of these, one case report, one original article, and two conference abstracts were excluded due to the duplication of data. Additional cases were diagnosed through our research project in 2017. The cases were reported between 1964 and 2017. Of the 24 case reports, twelve described Bangladeshi immigrants diagnosed overseas (UK, Belgium, USA, Cuba, and Kuwait), all of whom had a history of travelling to Bangladesh before their illness [5,6,7,8,9,10,11,12,13,14,15,16], whereas the rest were diagnosed and reported within Bangladesh [19,20,21,22,23,24,25,26,27,28,29,30,31].

### 2.2. Definitions of Clinical Manifestations:

In this review, major organ involvement and clinical features were classified as follows.

(a) Pulmonary: pneumonia, including complications such as a lung abscess or a pleural effusion.
(b) Musculoskeletal (MSK): septic arthritis, osteomyelitis, and others.
(c) Genitourinary (GU): infection of the urinary and genital tract, including the kidneys.
(d) Neurological: involvement of the brain and spinal cord, including the meninges.
(e) Organ abscess/deep-seated abscess: Abscess involving any solid organ or in any deep-seated site such as the muscles.
(f) Cutaneous: infection and abscess of the skin and subcutaneous tissue.
(g) Bacteraemia without focus: acute sepsis without any specific focus.

### 2.3. Climate Data

The monthly average mean temperature in degrees Celsius and the monthly average rainfall in millimetres for the years 1961 to 2015 were collected from the climate change knowledge portal of the World Bank [32].

### 2.4. Statistical Analysis

The results were expressed as median ± interquartile range (IQR) for continuous variables. A choropleth map was drawn using geographic information system (GIS) data to illustrate the distribution of the cases [33]. A choropleth map typically uses either differences in colour value (sometimes in combination with hue) or differences in spacing (e.g., the intensity of a hatched pattern) to represent the differences. The one sample *t*-test was applied to determine the level of significance. Statistical analysis was performed using IBM SPSS Statistics 22 for Windows, and GraphPad Prism 7 was used to display the results. Informed patient consent was obtained from the prospectively studied cases, with ethical permission being granted by the institutional committees of Dhaka Medical College (DMC) and the Bangladesh Institute of Research and Rehabilitation for Diabetes, Endocrine, and Metabolic Disorders (BIRDEM).

### 2.5. Important Findings

Between 1964 and 2017, 51 cases of culture-positive melioidosis were diagnosed from Bangladesh, with all the published cases listed in Table 1. The median age of the patients at presentation was 45 years (IQR 37–52), with a significant male (79.6%) predominance (Table 2). The oldest patient was 90 years old and the youngest was 8 years old. The median age of the patients in Bangladesh was slightly lower compared to India, Thailand, Taiwan, and Australia [34,35,36,37]. However, the male preponderance was higher than the findings from the same countries. A similar male predominance was found in Malaysia [38]. This is probably because in countries such as Bangladesh, males are more involved with outdoor activities. Moreover, the access to healthcare for women is still restricted due to many reasons, including cultural and socioeconomic factors in Bangladesh [39,40].

The majority of the patients for whom data was available (14/39; 36%) were farmers by profession, which is compatible with another hospital-based serosurveillance carried out in Bangladesh in 2010, where farmers had an increased risk of seropositivity (risk ratio = 1.4, 95% CI 1.0–1.8; *p* = 0.03) [41]. The survey recorded 28.9% positivity for *B. pseudomallei* through an indirect haemagglutination assay among 1244 adult febrile patients [41]. However, another study reported 22.6% to 30.8% seropositivity in three districts (Gazipur, Mymensingh, and Sylhet) where melioidosis cases were detected earlier, compared to 9.8% in a district (Kishoreganj) where no melioidosis cases were either detected or reported (*p* < 0.01) [42]. The same study found no significant difference among different occupational groups (χ^2^ = 3.835, *p* = 0.280) [42]. Over 88% (23/26) of cases in Bangladesh had a definitive history of soil exposure (Table 2). This is related to the occupational activities of the patients.

**Figure 1:**
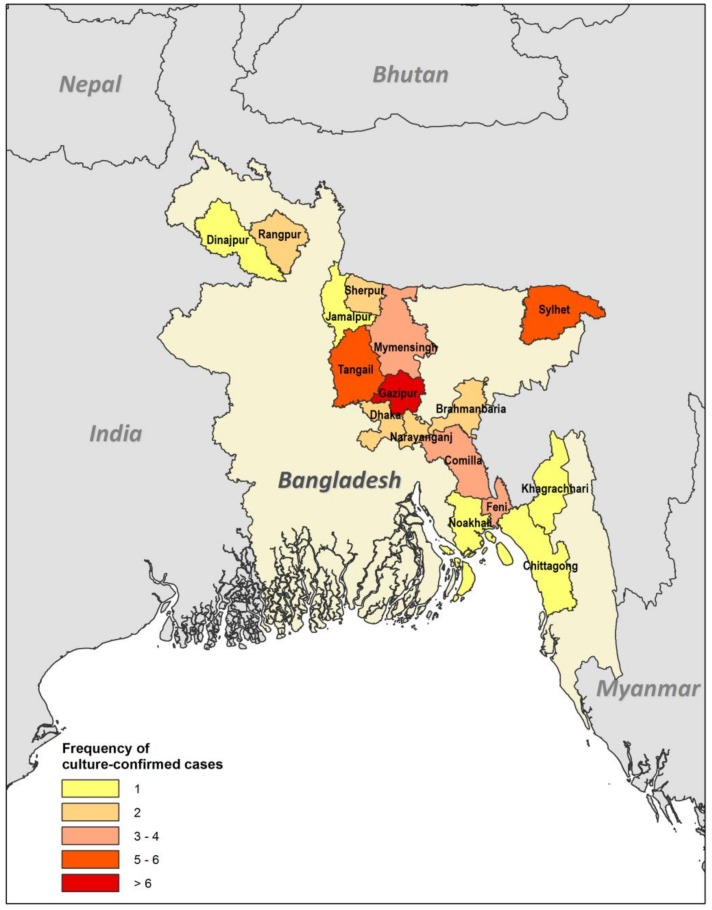
Choropleth map based on GIS data. The map illustrates the frequency by district of culture-confirmed cases of melioidosis (*n* = 46).

Cases have been reported from sixteen out of 64 districts of Bangladesh, all of which were in the eastern and northern parts of the country (Figure 1). In five patients, the geographic location could not be identified. The highest number of cases (9/46; 18%) were recorded from Gazipur, followed by Tangail (6/46; 12%), Sylhet (5/46; 10%), Mymensingh (4/46; 8%), Feni (4/46; 8%), and others. This study confirms that the Gazipur, Tangail, Sylhet, and Mymensingh districts are hotspots for melioidosis in Bangladesh. Our findings are in consensus with the soil surveillance carried out in these districts in 2011 and confirmed its presence. The isolates were phenotypically identical, arabinose negative and showed a specific 550 bp band in PCR [42]. The reason behind the dearth of cases to the west may be multiple. The agricultural pattern is regionally variable in Bangladesh, especially for rice, which is mainly grown in the north, south, and northeast districts [43]. Moreover, rice is cultivated in two seasons in the north, northeast, and south, but it is cultivated only once a year in the western districts [43]. There is also a clear variation in the pattern of rainfall. High rainfall occurs twice in the northeastern part of the country compared to the west [44]. However, the lack of cases in western Bangladesh could also be due to a lack of diagnosis and/or the underreporting of cases. The communication to the capital from the western and northwestern parts of the country is also more difficult compared to other regions. The infrastructure development index (IDI) is also very poor for those districts [45], so many patients may not travel to Dhaka and other major centres for diagnosis and treatment.

In thirty-eight cases, the period between the onset of symptoms and the diagnosis of melioidosis was calculated (Table 3). The median duration was 36 days (IQR 18.75–79.5). Specific risk factors/co-morbidities were identified in forty-eight cases. A high proportion (40/48; 83%) of the cases had diabetes mellitus (Table 3). Among these, 11 were already known to have diabetes at presentation and the rest (29; 76%) were diagnosed after hospital admission. The other comorbidities were smoking (3/48; 6%), chronic kidney disease (CKD; 2/48; 4%), and hypertension (2/48; 4%). We also found alcoholism, ischaemic cardiomyopathy (ICM), and haemoglobin E (HbE) trait in single cases. The presence of *B. pseudomallei* was confirmed in various culture samples. Twenty-three patients (45%) were positive on pus culture. The second most common positive sample was blood (22/51; 43%). Other sources of the bacterium were joint fluid (10/51; 20%), other swabs (6/51; 12%) such as skin, nasotracheal, and tracheal; urine (5/51; 10%) and sputum (3/51; 6%). In total, fourteen patients (27%) died and thirty-seven (73%) survived.

**Figure 2:**
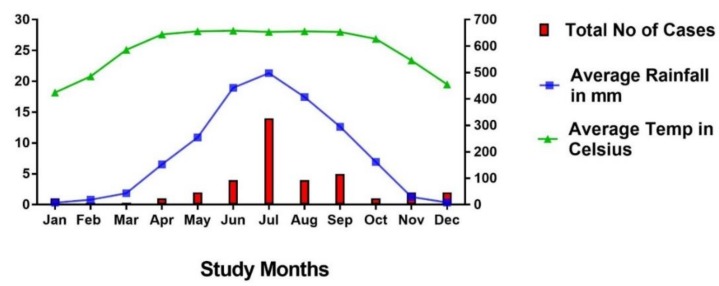
The monthly cases of culture-confirmed melioidosis, the average rainfall in mm, and the average temperature in degrees Celsius. Data represented from 1961 to 2015 [32].

The majority of cases were diagnosed during the rainy season from June to September (Figure 2). The primary clinical presentation and major system involvement of 49 cases are shown in Table 4. In two patients, there was insufficient information in the reports to enable their classification. A skin/soft tissue abscess was the most common primary clinical presentation (13/49; 27%), and none of the patients with localised cutaneous lesions died. The second most common presentation was septic arthritis (10/49; 20%), with one death occurring within this group of patients (a known diabetic woman with CKD who was admitted to hospital with shock and subsequently developed acute-on-chronic renal failure and died). The next most common presentations were pneumonia (7/49; 14%) and a deep-seated abscess/organ abscess (7/49; 14%). Two patients died of pneumonia and one with an organ abscess. Five patients (10%) presented with a urinary tract infection and/or acute kidney injury (AKI), and three of these patients died. All of these cases were admitted with shock and multiple organ involvement. Four (8.1%) patients presented with sepsis without any focus, and three (6%) presented with symptoms and signs suggesting a possible diagnosis of meningitis, although no lumbar punctures were done. All seven of these patients (median age 50 (IQR, 40–59)) were diabetic and developed multiple organ failure during admission and died.

In terms of treatment, 46.3% (19/41) patients were treated with ceftazidime and 41.4% (17/41) received meropenem. Among the cases diagnosed overseas and before 1999, four (9.7%) received cotrimoxazole plus chloramphenicol, and one patient (2.4%) received doxycycline plus amoxicillin/clavulanic acid as intensive phase therapy in this series.

## 3. Current Recommendations and Availability of Measures against Melioidosis

### 3.1. Surveillance Systems and Reporting

#### 3.1.1. Human

Melioidosis is not a statutorily notifiable disease in Bangladesh, and there is no formal surveillance system in place for human melioidosis. Reported cases therefore tend to arise from individual researchers who have a specific interest. At present, the health directorate (Ministry of Health and Family Welfare, Bangladesh) has no specific program or operational plan on melioidosis in Bangladesh.

#### 3.1.2. Animal

The infrastructure and facilities for veterinary services and research in Bangladesh are very weak. There is no surveillance system for animal melioidosis currently in place. No veterinary reports have ever been issued from Bangladesh.

### 3.2. Guidelines

Currently, Bangladesh does not have any national management or treatment guideline for melioidosis. The majority of indigenous cases were diagnosed and treated at BIRDEM General Hospital. The Microbiology Department of BIRDEM hospital follows the Clinical and Laboratory Standard Institute (CLSI) guidelines wherever possible. BIRDEM doctors follow current published treatment guidelines [46].

### 3.3. Treatment

The antimicrobial susceptibility pattern of clinical isolates showed 100% sensitivity to ceftazidime, imipenem, piperacillin–tazobactam, amoxicillin–clavulanic acid, and tetracycline by both disk diffusion and MIC methods in Bangladesh [47]. Intravenous antibiotics (ceftazidime and meropenem/imipenem) for the acute phase and oral antibiotics for the eradication phase are available in the local market (district and divisional level). Patients usually need to purchase their own drugs for the first two to three days of treatment, and thereafter, they are supplied by the hospital free of cost. It takes some time to order the drug, gain approval from the relevant authority, and obtain a supply from the hospital pharmacy. It is worth mentioning that a free supply of these drugs is only possible in tertiary level hospitals (medical college hospitals), and not in primary (subdistrict) or secondary (district) hospitals. In private clinics, patients need to bear the whole treatment cost. In a developing country such as Bangladesh, it is difficult for many to bear the treatment cost of melioidosis. There is also a dearth of intensive care unit (ICU) beds in tertiary level hospitals in Bangladesh. The moderately high mortality is probably due to late diagnoses, a delayed door-to-needle time for the delivery of appropriate antibiotics, and a lack of ICU care.

## 4. Awareness of Melioidosis

Awareness of melioidosis among clinicians, microbiologists, and health policymakers is inadequate. Although several case reports have been published in local and international journals, the probability of clinicians considering melioidosis as a diagnosis and requesting the laboratory to look specifically for the bacterium remains low. Public awareness of this organism, particularly among farmers and other high-risk populations, is very low. Recently, the Bangladesh Association for Advancement of Tropical Medicine (BAATM) and the Bangladesh Society of Medicine (BSM) took some special initiatives to raise awareness among clinicians by organising seminars and lectures. David A. B. Dance and Susanna J. Dunachie recently visited Bangladesh on their invitations and conducted seminars in Dhaka. The third South Asian Melioidosis Congress is planned to take place in Bangladesh in 2019, which will further contribute to raising awareness.

## 5. Major Achievements

So far, the scientists of Ibrahim Medical College and BIRDEM have conducted research on melioidosis in Bangladesh despite the relatively low numbers of cases reported. Doctors at BIRDEM have diagnosed the majority of cases so far in Bangladesh. However, apart from IMC and BIRDEM, microbiologists elsewhere in Bangladesh are unfortunately not currently diagnosing melioidosis. Therefore, we plan to organise capacity development workshops with the support of Mahidol Oxford Research Unit (MORU) and LaoOxford-Mahosot Hospital–Wellcome Trust Research Unit (LOMWRU) for the microbiologists and the clinicians working at tertiary level hospitals in Bangladesh, though funding is currently pending. We also recently completed a prospective study to see if melioidosis is a significant cause of febrile illness in Bangladesh at DMC and BIRDEM in collaboration with the University of Oxford, which will form the basis for a long-term study of melioidosis in Bangladesh.

## 6. Current and Future Challenges

The major challenges to the diagnosis and the treatment of melioidosis in Bangladesh are a lack of resources and a lack of awareness. Clinicians do not think about this disease in the first instance, and subsequently, microbiologists are not alerted to look specifically for the bacterium. In addition, microbiologists unfamiliar with *B. pseudomallei* are likely to discard the bacterium as a clinically insignificant environmental *Pseudomonas* species. Microbiologists other than those at the BIRDEM laboratory probably do not have the skills, training, and capacity to identify the organism. Therefore, diagnostic capacity development is the key area where immediate attention and funding is required. Awareness-building programs among clinicians are also necessary. Diabetes was found to be the most common risk factor for melioidosis in this series, and this was a similar observation elsewhere. The incidence of diabetes is rapidly increasing in Bangladesh, with 8.4 million people currently living with diabetes [48]. Therefore, we predict that melioidosis could become an enormous clinical challenge in Bangladesh. CKD, hypertension, and smoking were also identified as comorbidities in this series, which are also quite prevalent among Bangladeshi populations. Melioidosis infects both animals and humans. Surveillance among animal populations in the hotspot areas is also required to measure the burden. A One Health approach is needed for the successful mitigation and control of the problem.

## Figures and Tables

**Table 1:** Reports of all culture-confirmed melioidosis cases from Bangladesh.

Year of Report	Presumed Location of Infection in Bangladesh	Location of Diagnosis	No. of Case/Cases	Reference
1964	Chittagong	UK	1	5
1969	Unknown	UK	1	16
1970	Unknown	UK	1	15
1988	Unknown	Bangladesh	1	30
1991	Sylhet	UK	1	13
1999	Sylhet	UK	3	10
1999	Sylhet	UK	1	14
2000	Unknown	UK	1	12
2001	Sherpur	Bangladesh	1	31
2007	Unknown	Belgium	1	8
2007	Rangpur	Belgium	1	9
2012	Unknown	USA	1	7
2013	Unknown	Bangladesh	1	27
2014	Gazipur, Mymensingh, Tangail, Dhaka, Narayanganj, Khagrachari, Comilla	Bangladesh	15	23
2014	Gazipur	Bangladesh	1	26
2014	Unknown	Kuwait	1	6
2015	Gazipur	Bangladesh	1	19
2015	Gazipur	Bangladesh	1	21
2015	Mymensingh	Bangladesh	1	25
2015	Unknown	Bangladesh	1	24
2015	Gazipur	Bangladesh	1	28
2016	Brahmanbaria	Bangladesh	1	22
2016	Khagrachari	Bangladesh	1	29
2017	Unknown.	Cuba	1	11
2017	Narayanganj, Tangail, Feni, Comilla, Mymensingh, Dhaka, Noakhali, Jamalpur	Bangladesh	11	20

**Table 2:** Baseline demographic characteristics of the culture-confirmed cases.

Variables	Number (%)	*p* Value ^⍰^
Age (years; median, (IQR))	45 (37–52)	
Sex (*n* = 51)		
Men	41 (80)	0.0001
Women	10 (20)	
Occupation (*n* = 39) ^ψ^		
Farmer	14 (36)	
Housewife	8 (21)	
Worker/day labourer	4 (10)	
Unemployed	4 (10)	
Carpenter	2 (5)	
Others	7 (18)	
History of soil/environmental exposure (*n* = 26) ^ψ^		
Yes	23 (88)	0.0001
No	3 (12)	

^ψ^ Variables have missing data. ^⍰^ By one sample *t*-test.

**Table 3:** Prehospital and laboratory characteristics and clinical outcome of culture-confirmed cases.

Variables ^Ɨ^	Number (%)
Time between symptom onset and diagnosis (days; median, (IQR))	36 (18.75–79.5)
Risk factors (*n* = 48) ^ψ^	
Diabetes mellitus	40 (83)
Chronic kidney disease	2 (4)
Hypertension	2 (4)
Smoking	3 (6)
Others (alcoholism, ICM, Hb ET) ^€^	3 (6)
Culture-positive samples (*n* = 51) ^ψ^	
Blood	22 (43)
Pus	23 (45)
Joint fluid	10 (20)
Urine	5 (10)
Sputum	3 (6)
Other samples (skin/nasotracheal/tracheal aspirate)	6 (12)
Mortality (*n* = 51) ^ψ^	
Survived	37 (73)
Died	14 (27)

^Ɨ^ Variables have missing data. ^ψ^ includes multiple positive sites. ^€^ ICM—ischaemic cardiomyopathy, Hb ET—haemoglobin E trait.

**Table 4:** Clinical description of culture-confirmed melioidosis cases (*n* = 49).

Clinical Variables	Number (%)	Number of Deaths (%)
Primary Clinical Presentation		
Skin and subcutaneous abscess	13 (27)	0 (0)
Septic arthritis	10 (20)	1 (10)
Pneumonia	7 (14)	2 (29)
Organ abscess/deep-seated abscess	7 (14)	1 (14)
Urinary tract Infection/Acute kidney injury	5 (10)	3 (60)
Sepsis without focus	4 (8)	4 (100)
Meningitis	3 (6)	3 (100)
Major System Involvement ^ψ^		
Musculoskeletal	21 (43)	1 (5)
Organ abscess/deep-seated abscess	13 (27)	1 (8)
Cutaneous	13 (27)	0 (0)
Pulmonary	10 (20)	2 (20)
Genitourinary	9 (18)	3 (33)
Bacteraemia without focus	4 (8)	4 (100)
Neurological	3 (6)	3 (100)
Total		14 (27)

^ψ^ Includes multisystem involvement.
